# The accuracy of the Jamaican national physician register: a study of the status of physicians registered and their countries of training

**DOI:** 10.1186/1472-6963-8-253

**Published:** 2008-12-11

**Authors:** Jennifer Knight-Madden, Robert Gray

**Affiliations:** 1 Sickle Cell Unit, Tropical Medicine Research Institute, University of the West Indies, Kingston 7, Jamaica; 2 Department of Obstetrics, Gynaecology & Child Health, University of the West Indies, Kingston 7, Jamaica

## Abstract

**Background:**

The number of physicians per 10,000 population is a basic health indicator used to determine access to health care. Studies from the United States of America and Europe indicate that their physician registration databases may be flawed. Clinical research activities have suggested that the current records of physicians registered to practice in Jamaica may not be accurate. Our objective was to determine whether the Medical Council of Jamaica (MCJ) accurately records and reports the identities, number and specialty designation of physicians in Jamaica. An additional aim was to determine the countries in which these physicians were trained.

**Methods:**

Data regarding physicians practicing in Jamaica in 2005 were obtained from multiple sources including the MCJ and the telephone directory. Intense efforts at tracing were undertaken in a sub-sample of physicians, internists and paediatricians to further improve the accuracy of the data. Data were analysed using SPSS, version 11.5.

**Results:**

The MCJ listed 2667 registered physicians of which 118 (4.4%) were no longer practicing in Jamaica. Of the subset of 150 physicians who were more actively traced, an additional 11 were found to be no longer in practice. Thus at least 129 (4.8%) of the physicians on the MCJ list were not actively practising in Jamaica. Twenty-nine qualified physicians who were in practice, but not currently on the Jamaican register, were identified from other data sources. This yielded an estimate of 2567 physicians or 9.68 physicians per 10,000 persons. Seven hundred and twenty six specialists were identified, 118 from the MCJ list only, 452 from other sources, in particular medical associations, and 156 from both the MCJ list and other sources. Sixty-six percent of registered doctors completed medical school at the University of the West Indies (UWI).

**Conclusion:**

These data suggest that the MCJ list includes some physicians no longer practicing in Jamaica while underestimating the number of specialists. Difficulty in accurately estimating the number of practicing physicians has been reported in studies done in other countries but the under-reporting of the number of specialists is uncommon. Additional consideration should be given to strategies to ensure compliance with the annual registration that is mandated by law and to changing the law to include registration of specialist qualifications.

## Background

The number of physicians per 10,000 population is one of the basic health indicators used to determine access to health care [1] and it is the responsibility of each country to compile accurate, up to date health data. Increased availability of physicians is associated with improved health outcomes [2]. The Medical Council of Jamaica (MCJ) is mandated by legislation passed in 1972 to register all physicians practicing in Jamaica on an annual basis. Although a list of registered physicians is gazetted in the Jamaica Gleaner (a Jamaican newspaper), it is only accessible to those who purchase hard copies from the Government Printing Office, which has a single outlet in Kingston, the capital of Jamaica. Difficulties encountered by JK-M during efforts to contact physicians for clinical research using the MCJ database suggested that this list may not be accurate. Attempts to conduct a survey among doctors who would be expected to treat patients with asthma (internists, paediatricians and general practitioners) were severely hampered by the inability to locate these physicians. The legislation regarding registration of physicians was updated in 2004, to ensure the submission of evidence for continuing medical education [3]. Documentation of the area of specialization of physicians and the proportion of physicians who are specialists is also important to physician workforce planning. Although the MCJ facilitates the registration of specialists, it has no legislative authority to do so.

The accuracy of national physician databases has previously been questioned. Reports have suggested that the American Medical Association (AMA) Masterfile overestimated physician numbers [4-6]. The Physician License Registry of the Ministry of Health in Lithuania is updated every five years. As a result, Lovkyte et al had to utilise several data sources to review physician resources in that country [7]. Furthermore, there is a global shortage of health care workers [8]. Migration from less developed, poorer nations which have fewer physicians, to the developed world where remuneration is better, is common [8,9].

The University of the West Indies (UWI) commenced training medical students in Jamaica in 1948 as the University College of the West Indies (London) and this group of students graduated in 1954. The College obtained independent University status in 1962 and was the only medical school in the English-speaking Caribbean until 1989 when a second regional medical school was started at the Trinidad and Tobago campus of the UWI. Few Jamaicans train in Trinidad and Tobago, and few physicians trained there move to Jamaica to work. The UWI also began offering specialty training in paediatrics in 1972 and has since added many other specialty degrees.

The success of UWI in providing an adequate number of physicians to practice in Jamaica is dependent on the number of physicians needed, the number of physicians trained in Jamaica and the rate of migration of UWI medical graduates from Jamaica. This would be reflected in the proportion of UWI trained physicians that are registered annually. It is important, therefore, that appropriate workforce data are available to guide the governments and the university on the numbers of physicians and specialists that need to be trained. The aim of this study was to determine whether the MCJ accurately records and reports the identities, number and specialty designations of the nation's physicians. An additional aim was to determine what proportion of practicing physicians received their basic training at UWI.

## Methods

The 2005 MCJ list of registered physicians was obtained. Additional sources of information were used to locate additional physicians in practice. Requests for a list of all employed physicians were sent to the four Regional Health Authorities of which only one complied. Requests for membership lists were sent to all Jamaican medical associations and lists were provided by the Medical Association of Jamaica, the Association of General Practitioners, The Association of Surgeons, The Association of Radiologists, the Association of Consultant Physicians and the Paediatric Association of Jamaica. A list of all medical graduates from the UWI was also obtained. The Yellow Pages of the Jamaican telephone directory were examined to identify practicing physicians. In addition, the authors identified physicians who, to their certain knowledge, had migrated, retired or died. Physicians who had not died, retired or migrated were deemed to be currently practicing medicine in Jamaica. The study was approved by the Ethical Committees of the Faculty of Medical Sciences, UWI and the University Hospital of the West Indies (UHWI).

In order to maximize the accuracy of the data within the fiscal constraints of the study, intensive tracing of a sub-set of physicians was undertaken. A random sample of 150 internists, paediatricians and general practitioners, representing 5% of the total number of physicians identified from all sources, was chosen. Calls were made to all available numbers, visits were made to all available addresses and e-mail messages were sent to all available e-mail addresses.

## Results

### Physicians in practice

**Figure 1:**
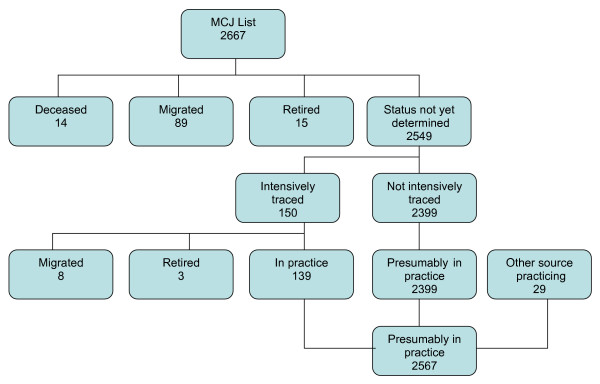
**Physicians listed as practicing in Jamaica**. Sources of data include the Medical Council of Jamaica list, a Regional Health Authority, Medical Associations, the telephone book and personal knowledge of the authors.

The MCJ listed 2667 registered physicians of which 118 (4.4%) were known to be no longer practicing in Jamaica (14 were deceased, 89 had migrated and 15 had retired). Of the subset of 150 physicians who were more actively traced, an additional 11 were found to be no longer practicing in Jamaica (8 had migrated and 3 had retired). Thus at least 4.8% of the physicians on the current MCJ list were no longer actively practicing in Jamaica (Figure 1). Furthermore, 29 qualified physicians who were in practice but not currently on the MCJ list were identified from the other data sources detailed above. This yielded a total of 2567 physicians or 9.68 physicians per 10,000 population, based on data from the Statistical Institute of Jamaica which estimated the Jamaican population in 2005 as 2,650,400[10].

During the process of identifying physicians, four persons were identified in the telephone book that purported to be physicians and were apparently practicing as such, but had never been licensed to do so. These individuals were reported to the Medical Council of Jamaica.

### Specialists

Seven hundred and twenty six specialists were identified, 118 from the MCJ list only, 452 from other sources only, in particular medical associations, and 156 from both the MCJ list and the other sources detailed above. The data are inadequate to allow calculation of the numbers of each particular specialty practicing in Jamaica.

### Medical training

**Figure 2:**
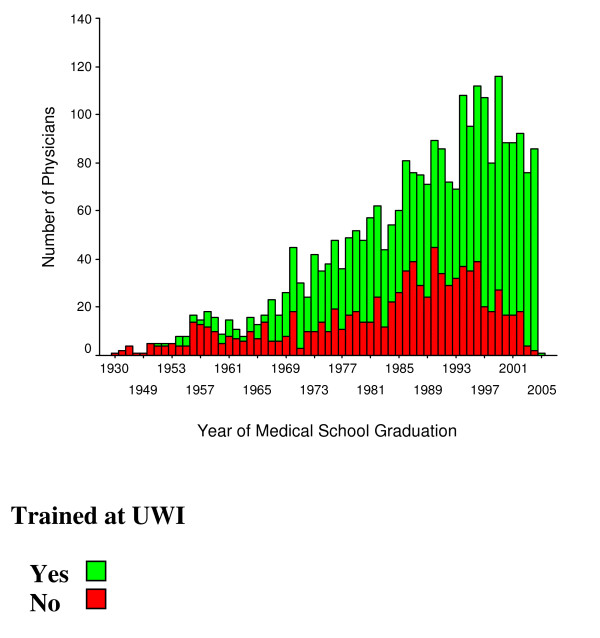
**Trends in source of training of physicians practicing in Jamaica, 1930–2006**. The source of data was the 2005 Medical Council of Jamaica list of physicians registered to practice in Jamaica.

Of the physicians on the 2005 MCJ list, 66% were trained in Jamaica, 10% obtained their medical degrees in India, 5% in Cuba and 4% in Myanmar (Table 1). The proportion of doctors registered annually who were trained at UWI increased significantly over time and by 1967 the primary site of basic medical training of registered doctors in Jamaica was the UWI (Figure 2). Of those doctors known to be specialists, 42% completed specialty training at UWI.

**Table 1:** Licensed physicians in Jamaica as reported by the Medical Council of Jamaica, by country of basic training, 2005

**Country/Region**	**Number of physicians**	**Percentage of physicians**
UWI	1756	65.8
India	263	9.9
Cuba	133	5.0
United Kingdom	119	4.5
Myanmar	111	4.2
USA	71	2.7
Nigeria	67	2.5
Russia	37	1.4
Other countries	110	4.1

Total	2667	100

## Discussion

The Medical Council of Jamaica's gazetted list of medical practitioners in 2005 did not adequately reflect all physicians registered to practice in Jamaica for several reasons. There were appropriately qualified physicians who were not registered and others who, although they were registered, had migrated or retired. Many physicians who migrate or retire do not notify the MCJ of their changed status. Despite the legislative requirement for an updated list, the MCJ had no active method of ascertaining changes in physician status. This has, to some extent, affected the accuracy of statistics about physician availability based on the MCJ list. Although the list compiled during this study was more accurate at the time of completion than the MCJ list, the trends seen in the subset of physicians that was more carefully traced suggest that this study may still have underestimated the total number of physicians who have migrated, retired or died. A limitation of the study was that financial constraints precluded in-depth tracing of all registered physicians. Full data were only available for the subset of 150 physicians, those known personally by the authors and those whose information was provided by a Regional Health Authority or medical association. The sub-sample, which included paediatricians, internists and general practitioners only, was not truly representative of the total population being studied. It does, however, demonstrate that the estimate of 9.68 physicians per 10,000 population may be a slight overestimate. If 7.3% of the 2667 registered physicians were no longer in practice in Jamaica, there would be 9.2 physicians per 10,000 population. For the year 2005, the estimated number of physicians in Jamaica in this report is similar to the estimate of 8.5 physicians/10,000 population in the Pan American Health Organisation [1].

The inadequacies in the data are not unique to the Jamaican situation. The lag in reporting in the AMA Masterfile, particularly with respect to the retirement of physicians, also caused overestimation of physician numbers [4]. Other authors have reported an overestimation of physician numbers by the AMA Masterfile when investigating its usefulness for identifying general practitioners and specialists. Some sources of data utilized in other settings are not available in Jamaica. Jamaica does not have a database from large health maintenance organizations [4]. Jamaican pharmacies rarely insist that physicians include their registration number on prescriptions [5]. The UWI Jamaica campus does not have easily accessible information regarding those who have obtained specialty certification [11]. In the absence of other sources of information, the accuracy of the MCJ list is even more important.

Contact information available to the MCJ was often outdated. Annual re-registration becomes due on the 1^st ^of January. In May 2006, more than half the doctors listed on the register had not re-registered for the year 2006[12]. A list of doctors who had complied with registration procedures was published in a Jamaican Gleaner newspaper during that year and so physicians who had not registered felt more pressured to comply with the regulations. The publication of this list and other active mechanisms, such as de-registration for non-compliance, need to be pursued to ensure annual registration of all physicians and compliance with the requirements for continuing medical education. Specific strategies need to be developed to identify those physicians who are retired or have died. This will enable the MCJ to publish a list that, like the AMA Masterfile, has fields denoting retired, deceased and inactive physicians.

In the Jamaica Gleaner of December 29, 2007, the Minister of Health was quoted as saying that doctors who were found to be practicing illegally in Jamaica would be disbarred from practice and prosecuted [13]. These measures should deter those few individuals who are not qualified physicians from attempting to practice as medical doctors. Although only four unqualified persons practicing fraudulently were identified, this study demonstrates that it is important that an up to date list of properly qualified doctors is easily accessible to the public.

The list of registered doctors also grossly underestimates the number of specialists currently practicing in Jamaica. Although physicians are encouraged to give information regarding specialization to the MCJ, it is clear that this is not commonly done. Information about specialists in practice is available from various medical associations but membership of these associations is entirely voluntary and it is estimated that fewer than one half of the registered physicians belong to any of them. This is in contrast to the report from Freed et al [11] which suggests that the AMA Masterfile may be overestimating the number of paediatric cardiologists practicing in the United States of America.

Accurate data collection regarding the number and level of training of physicians is important for several reasons. It would allow for comparison with other countries, help Government to determine whether it should actively recruit personnel abroad and stimulate UWI to encourage medical graduates to obtain further training in specialty areas that are deficient in Jamaica.

When compared with the summary statistics available for other countries in the region, the estimate based on these data of 9.7 doctors per 10,000 population in Jamaica which, while falling below the average for the Latin American and Caribbean Region (18.3 physicians per 10,000 population), is similar to the average for the English Speaking Caribbean area (8.5 physicians per 10,000 population)[1]. However efforts need to be made to improve the data collection regarding physician status in Jamaica before this estimate is deemed to be accurate.

There are international examples of systems which make data more easily available to the general public. In the United Kingdom, registration with the General Medical Council (GMC) is not only required for physicians to practice within the country, but also for specialists to practice within their designated specialty GMC [14]. Any member of the public in that country may access information concerning the registration status of their physician [14]. General practitioners must register with the state in the United States of America and with the province in Canada and registration information regarding specific physicians, localities or specialties is available on line to the general public in both countries [15,16]. In the United States of America, Specialty Boards record all those who qualify across the country in their particular specialties [17] but these data are sometimes not in agreement with the AMA Masterfile [11]. In Canada, the Royal College of Physicians and Surgeons lists all specialists trained in that country [18]. The process of establishing a specialist register is complex but guidance on this may be obtained from Malaysia which has recently embarked on this exercise [19] and from the Caribbean Association of Medical Councils. In addition, the International Association of Medical Regulatory Authorities is a likely source of useful assistance [20]. Since the UWI is playing a pivotal role in the training of both general practitioners and specialists who work in Jamaica, it would be better informed about areas of need and surfeit regarding specialty training if there was a specialist register.

Recommendations arising from this study include

(1) Punitive action against physicians who do not provide the annually required data for registration

(2) Regular publication of the list of registered doctors in the print media

(3) Establishment of a web-site that provides access to the current list of registered doctors and specialists with their contact data and that facilitates the process of registration on line

(4) Amendment of legislation to include the requirement for specialists to provide proof of specialist qualification to the MCJ before being allowed to advertise themselves as specialists.

## Conclusion

These data suggest that the MCJ list may overestimate the number of physicians and significantly underestimates the number of specialists practicing in Jamaica. Additional strategies need to be identified to allow these data to more accurately reflect the numbers and status of physicians practicing in Jamaica.

## Abbreviations

MCJ: Medical Council of Jamaica; UHWI: University Hospital of the West Indies; UWI: University of the West Indies.

## Competing interests

The authors declare that they have no competing interests.

## Authors' contributions

JK-M conceived and designed the study, coordinated the acquisition of data, completed the analysis and interpretation of data, drafted the manuscript and has given final approval of the version to be published.

RG made substantial contributions to the acquisition and interpretation of data; has revised the manuscript critically for important intellectual content; and has given final approval of the version to be published.

## Pre-publication history

The pre-publication history for this paper can be accessed here:


